# Cross-sectional association of oxidative balance score with cataract among US adults: NHANES 1999–2008

**DOI:** 10.3389/fnut.2025.1555631

**Published:** 2025-03-25

**Authors:** Ning Li, Yuchen Fan, Juan Li, Juanjuan Guo, Jianfeng Wang, Ziqing Gao

**Affiliations:** Ophthalmology Department‌, The First Affiliated Hospital of Bengbu Medical University, Bengbu, Anhui, China

**Keywords:** NHANES, oxidative balance score, cataract, oxidative stress, antioxidant-rich diet

## Abstract

**Objective:**

Oxidative stress plays a crucial role in the onset and progression of cataracts. As a comprehensive indicator of an individual’s oxidative stress status, OBS integrates dietary antioxidant intake and lifestyle factors, providing a holistic assessment of oxidative-antioxidative balance. However, research on the association between OBS and cataracts remains limited. Therefore, our study aims to bridge this research gap and provide novel epidemiological evidence supporting the role of oxidative stress in cataract prevention.

**Methods:**

A total of 13,409 subjects from the National Health and Nutrition Examination Survey (NHANES) conducted between 1999 and 2008 were selected. The OBS was calculated based on 16 dietary factors and 4 lifestyle factors. Weighted logistic regression and restricted cubic splines (RCS) were employed to assess the association between OBS and cataract.

**Results:**

The prevalence of cataract was found to be 12.2%. The restricted cubic spline analysis did not support a non-linear association between OBS and the prevalence of cataract (*p* = 0.742). After categorizing participants into quartiles based on OBS, those in the Q4 group exhibited lower odds of developing cataract (OR: 0.827, 95% CI: 0.713, 0.958, *p* < 0.01) compared to the Q1 group. Subgroup analysis revealed that significant associations were observed only among males, individuals with an education level below high school, those with a poverty income ratio (PIR) ranging from 1.3 to 3.49, and individuals with a Charlson comorbidity index (CCI) of 2 or higher.

**Conclusion:**

The OBS demonstrated a strong negative correlation with cataract prevalence. These results underscore the importance of adhering to an antioxidant-rich diet and lifestyle for cataract prevention, as well as the need to consider individual and population-specific factors in future research and prevention strategies.

## Introduction

Cataracts present a substantial public health concern around the world, which characterized by the loss of transparency of the lens, are a prevalent cause of visual impairment and blindness among the elderly globally (1). The World Health Organization reports that nearly 180 million individuals worldwide experience visual impairment, with cataracts being a contributing factor, with cataracts contributing to 46% of these cases (2). Aging is the leading cause of cataracts, other lifestyle and environmental factors such as smoking, alcohol consumption, and prolonged exposure to sunlight are also significantly influence factors (3, 4).

Currently, no effective pharmacological treatment has been proven to reverse or halt cataract progression. Existing medications, such as antioxidant eye drops (e.g., glutathione, vitamin C, *N*-acetylcysteine), have demonstrated antioxidant effects *in vitro* and in certain animal studies (5, 6). However, large-scale clinical trials have not consistently confirmed their efficacy. Moreover, the long-term benefits of these drugs remain uncertain, and patient adherence is often poor. Therefore, preventive strategies are of paramount importance. While cataract surgery can substantially improve the vision (7), its accessibility is often limited by economic and surgeons.

Antioxidants, as a key component of dietary and lifestyle interventions, have been extensively studied and are increasingly recognized for their role in reducing oxidative stress and delaying cataract onset and progression.

Oxidative stress has been widely recognized as a key contributor to cataract development (8, 9). Prolonged oxidative stress leads to the oxidation of lens proteins and lipids, resulting in protein aggregation and loss of lens transparency (10). Studies have suggested that antioxidant intake may mitigate oxidative damage and potentially reduce the risk of cataracts (11, 12). Given the critical role of oxidative stress in cataract pathogenesis and its potential modifiability through diet and lifestyle, this study focuses on the association between oxidative balance score (OBS) and cataract risk.

The OBS is a nuanced indicator for evaluating oxidative homeostasis in individuals, was derived by counting the antioxidant and pro-oxidant components of dietary and lifestyle factors (13). An elevated OBS is often associated with unhealthy dietary and lifestyle practices that may increase the risk of cataracts. While oxidative stress is known to play a significant role in the pathogenesis of cataracts, current studies do not provide sufficient evidence to establish a definitive relationship between the OBS and cataracts. This study aims to explore this relationship based on data from the National Health and Nutrition Examination Survey (NHANES) conducted between 1999 and 2008.

## Materials and methods

### Study participants

NHANES project utilizes a complex, multi-stage sampling strategy to evaluate the health and nutritional status of the U.S. population‌ conducted by the National Center for Health Statistics (NCHS) and the Centers for Disease Control and Prevention (CDC). The survey encompasses various aspects of health and lifestyle, including demographic, dietary, medical, and environmental factors, providing invaluable data for researchers, health professionals, and policymakers. Ethical approval for NHANES has been granted by the Ethics Review Board of the National Center for Health Statistics, and all participants have provided written informed consent.

**Figure 1:**
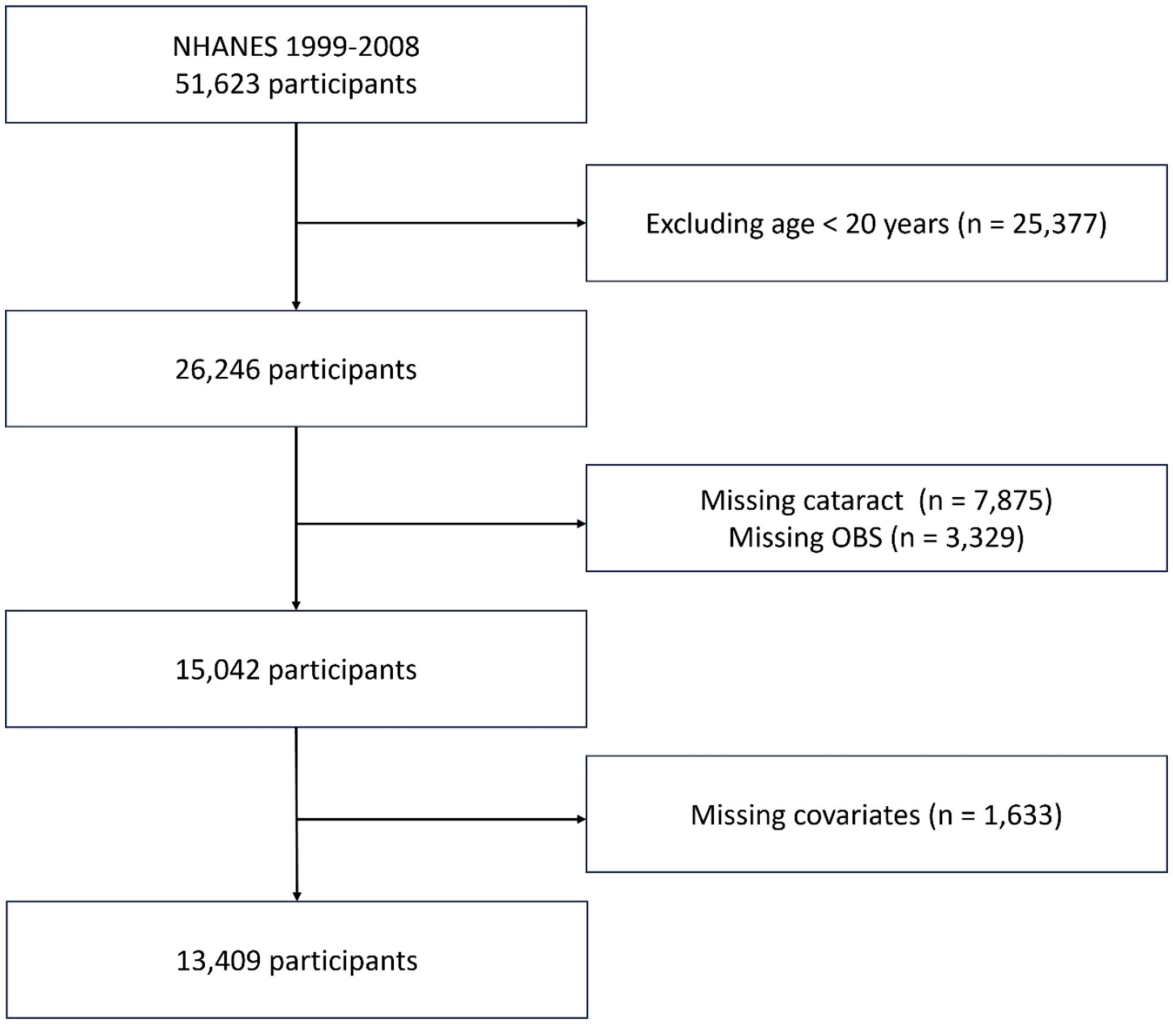
Selection process of study participants.

In this study, we utilized data from the NHANES database, spanning five consecutive cycles from 1999 to 2008, specifically: 1999–2000, 2001–2002, 2003–2004, 2005–2006, 2007–2008. ‌Exclusion criteria for this study: (1) aged less than 20 years, (2) missing information on cataract, (3) missing data on OBS, and (4) missing covariates data‌. After applying these criteria, ‌a total of 13,409 survey participants were included in our analysis (Figure 1).

### Assessment of oxidative balance score

The OBS was assessed utilizing ‌a combination of 16 dietary factors and 4 lifestyle factors, which encompassed 15 antioxidants and 5 pro-oxidants‌. The 15 antioxidants comprised dietary fiber, carotene, riboflavin, niacin, vitamin B6, total folate, vitamin B12, vitamin C, vitamin E, calcium, magnesium, zinc, copper, selenium, and physical activity‌. These antioxidants were scored on a scale of 0, 1, and 2, representing low, moderate, and high levels, respectively. ‌The 5 pro-oxidants included total fat, iron, alcohol consumption, BMI, and cotinine levels‌, which were scored on a scale of 0, 1, and 2, representing high, moderate, and low levels, respectively. ‌OBS was defined as the cumulative sum of all the points obtained from these factors‌.

### Cataract diagnosis

Cataract diagnosis was determined based on self-reported history of cataract surgery, which served as a proxy indicator. Participants were asked, ‘Have you ever had a cataract operation?’ (VIQ070/VIQ071). Those who responded ‘yes’ were classified as having been diagnosed with cataract. Since NHANES is a cross-sectional survey, it does not provide detailed clinical history, such as the onset, severity, or progression of cataracts before surgery. Therefore, our study primarily captures individuals who have undergone cataract surgery rather than those with undiagnosed or early-stage cataracts.

### Potential covariates

We incorporated several potential covariates to adjust our models based on prior knowledge. ‌Age‌ was categorized as ‌<65 and ≥65‌. ‌Sex‌ was classified as ‌male and female‌. For ‌education‌, we considered ‌less than high school, high school diploma, and more than high school‌. ‌Race‌ was divided into ‌non-Hispanic white, non-Hispanic black, and Hispanic/other‌. The ‌poverty index‌ was stratified into ‌<1.3, 1.3 ~ 3.49, and ≥3.5‌. Additionally, we included the ‌Charlson comorbidity index (CCI)‌, which was calculated by summing up scores for various reported diseases‌. Participants who did not report a specific disease were assigned a score of 0 for that disease.

### Statistical analysis

Due to the complex multistage cluster survey design of NHANES, the samples were assigned weights. ‌Continuous variables with normal distribution were presented as weighted means and standard deviations (SD), while categorical variables were expressed as relative numbers and weighted percentages. Chi-square tests were employed to compare differences of basic characteristics according to cataract or not. A weighted logistic regression model was used to investigate the associations between OBS and the prevalence of cataract. Which were showed as odds ratio (OR) and 95% confidence interval (CI). ‌Three models were constructed: Model 1 was unadjusted; Model 2 adjusted for age and sex; ‌Model 3 further adjusted for education, race, poverty index, and Charlson comorbidity index. OBS was initially analyzed as a continuous variable. Subsequently, OBS was divided into four quartile groups (Q1, Q2, Q3, Q4). A restricted cubic spline (RCS) was applied to test the non-linear relationships between OBS and cataract. Subgroup analyses by basic characteristic were performed to determine‌ ‌susceptible population‌.

Statistical significance was set at 0.05. All analyses were conducted using R v4.2.2 (The R Foundation, Vienna, Austria).

## Results

### Basic characteristics of study participants

The study involved 13,409 participants‌, comprising ‌6,581 males (47.77%) and 6,828 females (52.23%)‌. The ‌mean age of the participants was 55.4 ± 17.8 years‌. One thousand, six hundred thirty-seven cataract participants‌ were identified, with the prevalence of 12.2%. Table 1 showed the distribution of baseline characteristics according to cataract status. Notably, ‌individuals with higher prevalence of cataract were more likely to be older (>=65 years), female, have a lower education level, be non-Hispanic white, have a lower poverty index, and have a higher CCI.

**Table 1:** The basic characteristics of study participants according to cataract status.

Characteristics	Total	Non-cataract	Cataract	*P*
Age group				<0.01
<65	8,755 (75.53)	8,555 (81.27)	200 (16.72)	
> = 65	4,654 (24.47)	3,217 (18.73)	1,437 (83.28)	
Sex				<0.01
Male	6,581 (47.77)	5,824 (48.62)	757 (38.97)	
Female	6,828 (52.23)	5,948 (51.38)	880 (61.03)	
Education				<0.01
Less than high school	4,205 (19.90)	3,529 (18.71)	676 (32.09)	
High school diploma	3,149 (24.96)	2,782 (24.87)	367 (25.94)	
More than high school	6,055 (55.14)	5,461 (56.43)	594 (41.97)	
Race				<0.01
Non-Hispanic white	7,136 (75.03)	5,988 (73.98)	1,148 (85.77)	
Non-Hispanic black	2,500 (9.60)	2,316 (10.00)	184 (5.57)	
Hispanic/Other	3,773 (15.37)	3,468 (16.02)	305 (8.66)	
Poverty index, *n*				<0.01
< 1.3	3,669 (18.07)	3,176 (17.61)	493 (22.80)	
1.3 ~ 3.49	5,280 (36.59)	4,505 (35.33)	775 (49.52)	
> = 3.5	4,460 (45.34)	4,091 (47.06)	369 (27.68)	
Charlson comorbidity index				<0.01
0	8,908 (71.43)	8,208 (73.97)	700 (45.43)	
1	1,670 (9.82)	1,389 (9.29)	281 (15.17)	
> = 2	2,831 (18.75)	2,175 (16.73)	656 (39.40)	

### Oxidative balance score and cataract

Table 2 showed the results of weighted logistic regression model on the association between OBS and the odds of cataract. In the unadjusted model, compared to OBS Q1 group, participants in Q3 and Q4 group had a lower odds of cataract (Q3 vs. Q1: OR = 0.758, 95%CI: 0.624, 0.920; Q4 vs. Q1: OR = 0.562, 95%CI: 0.487, 0.649). In the fully adjusted model by age, sex, education, race, poverty index, CCI, participants in Q4 group had a lower odds of cataract (OR: 0.827, 95%CI: 0.713, 0.958) compared to OBS Q1 group.

**Table 2:** Weighted logistic regression on the association between OBS and the odds of cataract.

	Model 1	Model 2	Model 3
OBS (Continuous)	0.972 (0.965, 0.979)	0.987 (0.979, 0.995)	0.991 (0.983, 0.999)
OBS (Quartiles)			
Q1 (Reference)	1	1	1
Q2	0.923 (0.774, 1.101)	1.011 (0.851, 1.201)	1.016 (0.852, 1.211)
Q3	0.758 (0.624, 0.920)	0.887 (0.726, 1.085)	0.919 (0.747, 1.131)
Q4	0.562 (0.487,0.649)	0.771 (0.670, 0.888)	0.827 (0.713, 0.958)
*P* for trend	<0.01	<0.01	<0.01

Model 1: Unadjusted model.

Model 2: Adjusted for age, sex.

Model 3: Adjusted by age, sex, education, race, poverty index, Charlson comorbidity index.

### Non-linear association of oxidative balance score with cataract

**Figure 2:**
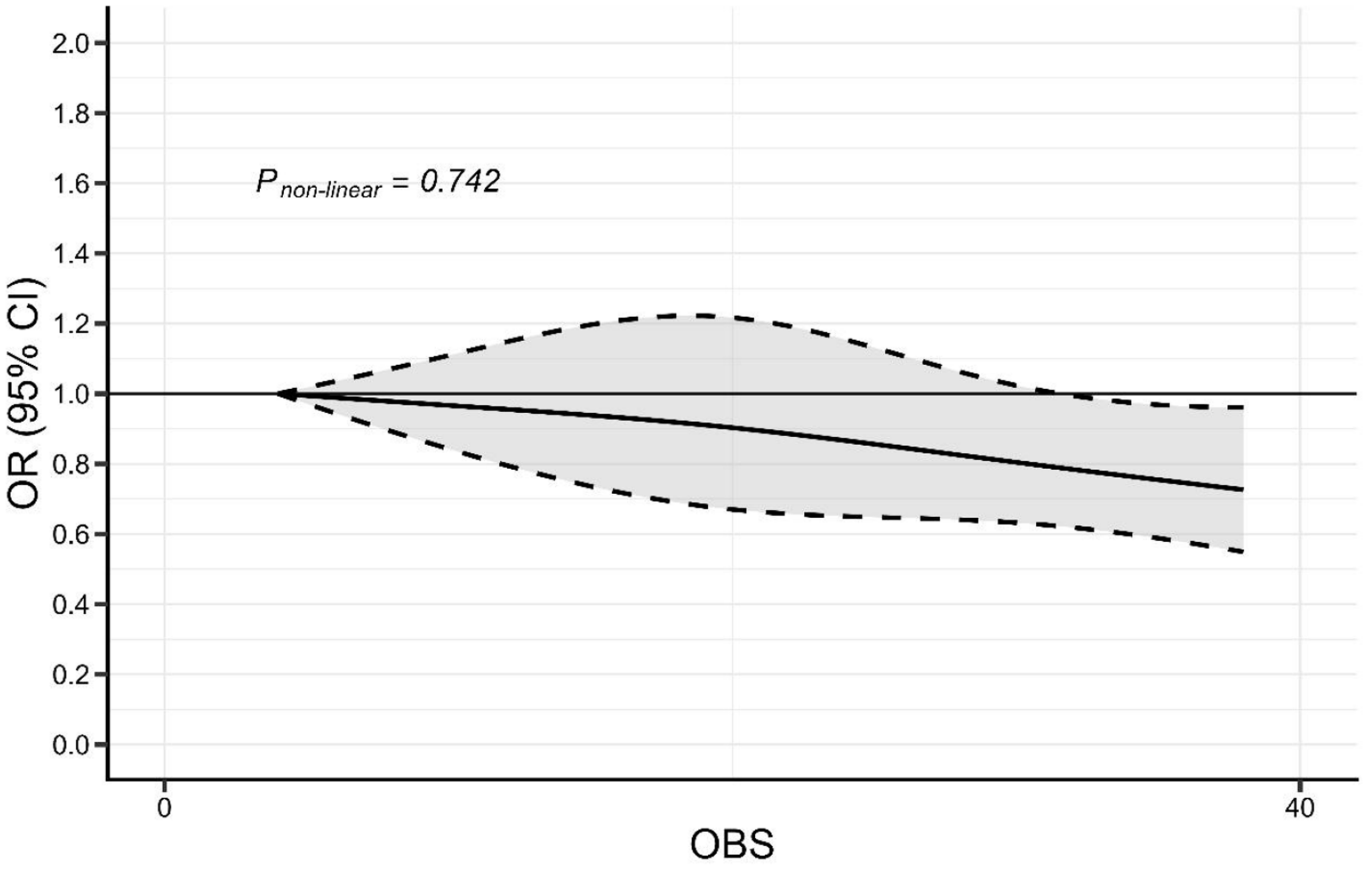
Spline curves showing the non-linear association of OBS with the prevalence of cataract (the solid line represents the adjusted OR, while the shaded area indicates the 95% CI).

As shown in Figure 2, restricted cubic spline did not support a non-linear association of OBS and the prevalence of cataract, with a non-linear *p* value of 0.742. Higher OBS was associated with a lower odds of the prevalence of cataract.

### Subgroup analysis

Table 3 displayed the subgroup analyses examining the relationship between OBS and the odds of cataract across various basic characteristic subgroups. Though, the interaction effect was not statistically significant, ‌significant associations were only observed among males, individuals with an education level less than high school, those with a PIR ranging from 1.3 to 3.49, and individuals with a CCI of 2 or higher‌.

**Table 3:** Subgroup analyses on the association between OBS and cataract.

Subgroups	OR (95% CI)	*P*-interaction
Age group		0.228
<65	0.979 (0.954, 1.005)	
> = 65	0.994 (0.985, 1.004)	
Sex		0.163
Male	0.981 (0.968, 0.994)	
Female	0.997 (0.986, 1.008)	
Education		
Less than high school	0.985 (0.970, 1.000)	
High school diploma	0.989 (0.974, 1.004)	0.922
More than high school	0.995 (0.980, 1.011)	0.422
Race		
Non-Hispanic white	0.992 (0.983, 1.000)	
Non-Hispanic black	0.998 (0.973, 1.024)	0.775
Hispanic/Other	0.976 (0.943, 1.011)	0.443
Poverty index, *n*		
< 1.3	1.001 (0.981, 1.020)	
1.3 ~ 3.49	0.984 (0.970, 0.999)	0.340
> = 3.5	0.994 (0.973, 1.016)	0.668
Charlson comorbidity index		
0	0.995 (0.981, 1.009)	
1	1.011 (0.981, 1.041)	0.258
> = 2	0.977 (0.964, 0.991)	0.075

## Discussion

Previous studies have demonstrated that oxidative stress is linked to the pathogenesis of cataracts, resulting from an imbalance between ROS and intracellular antioxidant defenses. Oxidants such as super-oxide anions, hydrogen peroxide, and hydroxyl radicals disrupt the body’s antioxidant defenses, which include enzymatic antioxidants (e.g., super-oxide dismutase and glutathione per-oxidase) and non-enzymatic antioxidants (such as vitamin C, vitamin E, and beta-carotene). This disruption leads to excessive production of oxidants, which in turn causes protein damage, apoptosis, and the subsequent formation of cataracts (13, 14). Although there is a strong link between oxidative stress and the progression of cataracts, there is still a lack of extensive epidemiological studies examining the connection between oxidative stress and the onset of cataracts. Although our research represents a significant advancement in addressing this gap, we acknowledge that it is a cross-sectional assessment and does not allow for direct validation of the underlying pathological processes. To further investigate the relationship between OBS and cataract, we conducted a cross-sectional analysis of 13,409 individuals within the NHANES cohort. The results indicated that in fully adjusted multivariate analyses, there was a significant negative association between OBS and cataract incidence, suggesting that subjects with healthier dietary patterns were less likely to develop cataracts. Further analysis, including propensity score adjustments, regression analyses, non-linear tests, and subgroup evaluations, reinforced this finding.

We assigned numerical values to 16 dietary components (14 antioxidants and 2 pro-oxidants) and 4 lifestyle factors (1 antioxidant and 3 pro-oxidants) to determine the OBS. Our findings highlight the protective effect of a higher OBS on cataracts and underscore the importance of an antioxidant-rich diet and lifestyle in reducing cataract risk. This observation is consistent with existing knowledge regarding the effects of an antioxidant-rich diet and lifestyle on cataracts (15–18). The role of dietary nutrients in eye health has been confirmed by researchers who emphasize the significance of carotenoids and antioxidants (19), which exhibit protective effects against macular degeneration and cataracts. However, prospective investigations of dietary patterns for cataract prevention remain limited, previous cross-sectional and retrospective studies have reported that the Healthy Eating Index, a healthy eating pattern proposed by Dietary Guidelines for Americans is associated with a reduced risk of cataracts (20). Moreover, a functional food (FF) mixture containing an aldose reductase inhibitor and an anti-glycation bioactive compound (21) has been shown to reduce lens opacification and delay cataract progression in a diabetic rodent model.

The results of our study also highlight sex differences in the risk of cataract development, indicating that women are at a higher risk than men, women may derive greater benefits from dietary and lifestyle modifications aimed at preventing and treating cataracts. Previous studies suggest that this gender difference may be attributed to several factors. Firstly, postmenopausal estrogen decline in women may lead to increased oxidative stress and inflammatory responses, which could accelerate lens aging and cataract formation (22, 23). Secondly, women generally have a longer life expectancy than men, and the risk of cataract increases with age, making women more likely to develop cataracts over their lifetime (24, 25). Additionally, differences in access to healthcare and health management practices may contribute to the observed disparity. There is increasing evidence worldwide that lower socioeconomic status (education, employment, and income) is linked to both the prevalence and progression of cataracts (26), which aligns with our findings. A prospective cohort study indicated that higher education levels correlate with a reduced risk of cataracts (27). Both income and education levels were inversely related to the 10-year cumulative incidence of nuclear cataracts from the Beaver Dam Eye Study (28). Similar trends have been observed in studies conducted in many other countries (29–31), such as Iran, Australia, India and China. Several explanations may account for the higher prevalence of cataracts among individuals of low socioeconomic status. First, these individuals often lack the financial resources necessary to afford cataract surgery. In comparison to those with higher socioeconomic status, individuals in this group may be influenced by various lifestyle factors, including smoking, alcohol consumption, physical activity, and diet quality. Moreover, environmental exposures, such as sunlight, indoor cooking smoke and outdoor occupations may also play a significant role in cataract development (32). Our study suggests that the association between OBS and cataracts is more pronounced among individuals with at least a high school education owing to the fact that individuals with higher education levels generally possess greater economic resources and a heightened awareness of dietary choices compared to those less-educated counterparts. Furthermore, we observed a significant correlation between OBS and CCI, which may be attributed to the negative association between OBS and various diseases, including diabetes mellitus (33), hyperuricemia (34), rheumatoid arthritis (35) and chronic kidney disease (36). Therefore, a higher CCI is associated with a higher OBS.

The benefits of this research include its unique topic, substantial sample size, and comprehensive statistical methods. Nonetheless, the present study does exhibit certain limitations. First, as a cross-sectional study, it cannot establish a causal relationship between OBS and cataract risk. Future prospective cohort studies are needed to validate our findings. Second, the calculation of OBS is based on dietary and lifestyle factors, which may not comprehensively capture all contributors to oxidative balance, such as genetic predisposition or environmental exposures. Future research incorporating genetic or exposomic analyses could provide deeper insights into the underlying mechanisms. Additionally, while subgroup analyses were performed, differences across specific populations (e.g., occupational groups) were not further explored. Future studies could focus on more refined population classifications to offer targeted cataract prevention strategies.

## Data Availability

The datasets presented in this study can be found in online repositories. The names of the repository/repositories and accession number(s) can be found at: https://wwwn.cdc.gov/nchs/nhanes/.
